# The effect of hybridization of *Culex pipiens* complex mosquitoes on transmission of *West Nile virus*

**DOI:** 10.1186/1756-3305-6-305

**Published:** 2013-10-23

**Authors:** Alexander T Ciota, Pamela A Chin, Laura D Kramer

**Affiliations:** 1 The Arbovirus Laboratories, Wadsworth Center, New York State Department of Health, Slingerlands, NY 12159, USA; 2 School of Public Health, SUNY, Albany, NY 12201, USA

## Abstract

**Background:**

*Culex pipiens* L. complex mosquitoes have a global distribution and are primary vectors of pathogens of public health significance. In the U.S., *Cx. pipiens* bioformes, *Cx. pipiens* form pipiens and *Cx. pipiens* form molestus, as well as *Cx. quinquefasciatus*, are primary vectors of *West Nile virus* (WNV; *Flaviviridae, Flavivirus*). These mosquitoes reside in distinct but overlapping ecological niches and readily hybridize in areas where they coexist. Although species and population-specific differences in vector competence of *Culex* mosquitoes for WNV have been identified, the extent to which hybridization within this complex alters WNV transmission potential has not been well characterized.

**Findings:**

WNV vector competence of laboratory colonies of *Cx. p.* f. pipiens*, Cx. p.* f. molestus, and *Cx. quinquefasciatus* was assessed and compared to hybrid populations created from reciprocal mating of these lines. The results demonstrate that hybridization has a significant effect on WNV infection, dissemination, and, particularly, transmission in *Culex pipiens* L. complex mosquitoes. Specifically, enhanced transmission of WNV was measured in all hybrid populations relative to one or both parental stains.

**Conclusion:**

These findings demonstrate that environmental or anthropogenic changes resulting in fluctuations in the distribution and extent of hybrid populations of *Culex* mosquitoes could have a significant impact on transmission patterns of WNV in nature.

## Findings

### Introduction

The *Culex pipiens* L. complex includes *Cx. pipiens* and *Cx. quinquefasciatus* in North America, South America, Africa, and Asia; as well as *Cx. australicus* and *Cx. globocoxitus* in Australia [1]. Mosquitoes in this complex are primary vectors of *West Nile virus* (WNV; *Flaviviridae, Flavivirus*) in the United States, i.e., *Cx pipiens* north of 36° latitude and *Cx quinquefasciatus*, south [2,3], with hybrids of the two found in a zone stretching from approximately 30°N to 40^o^N latitude in N. America [4,5]*.* Each species possesses a unique genetic signature as well as distinct physiology [6,7]. *Cx pipiens* is comprised of two bioformes*, Cx pipiens* form pipiens and *Cx pipiens* form molestus. *Cx. p.* f. molestus populations are present throughout the Americas where they are most often found in more subterranean areas, whereas *Cx. p.* f. pipiens and *Cx. quinquefasciatus* occupy aboveground habitats. Additional biological differences further distinguish *Cx. p.* f. molestus, including autogeny, mating in enclosed spaces, and lack of diapause [2,8]. An excellent review of the *Cx. pipens* complex has been published elsewhere [1]. Although many Northern European populations of *Cx. p.* f. pipiens are pure, U.S. populations generally contain varying levels of *Cx. p.* f. molestus signature, a characteristic which may increase propensity for U.S. *Cx. pipiens* to feed on mammals and contribute to the increased number of human cases of WNV in the U.S. [9]. Vector competence for WNV also has been shown to vary among *Culex* species and populations [10,11], yet the extent to which hybridization within this complex alters WNV transmission has not been fully evaluated. Here, we sought to characterize variation in WNV transmission potential in laboratory colonies of *Cx. p.* f. pipiens*, Cx p.* f. molestus, and *Cx. quinquefasciatus*, as well as hybrids resulting from mating of these parental lines. Our results demonstrate that the extent of hybridization among *Cx. pipiens* complex mosquitoes may significantly alter patterns of WNV transmission in the U.S.

### Methods

#### Mosquitoes

All colonized *Culex* mosquitoes were maintained in 30.5 cm^3^ cages in an environmental chamber at 27+/-2°C with a relative humidity of 45-65% and a photoperiod of 16:8 (light:dark) hours prior to the collection of experimental egg rafts. *Cx. p.* f. pipiens colony mosquitoes were originally collected in Pennsylvania in 2004 (courtesy of M. Hutchinson) and colonized at the Arbovirus laboratory. *Cx. quinquefasciatus* colony mosquitoes were derived from a laboratory colony provided by D. Fonseca (Rutgers Univ.) derived from egg rafts from Benzon Research Inc. (Carlisle, PA) originating from a highly colonized US population. *Cx. p.* f. molestus were colonized in 2009 following collection from the basement of the state house in New Jersey (courtesy of D. Fonseca). Two-way crosses were completed with the 3 parental strains to produce hybrid progeny. Genetic signatures were confirmed using species-specific primers on a subset (10 individuals) of F0 and F1 mosquitoes [12,13]. Parental *Cx. p.* f. pipiens were confirmed to be genetically distinct from *Cx. quinquefasciatus* yet, as expected, did contain *Cx. p.* f. molestus signature. *Cx. quinquefasciatus* and *Cx. p.* f. molestus individuals tested were genetically pure at the loci evaluated and the presence of mixed signatures were confirmed among all F1 hybrid populations used for experimental feedings.

#### Experimental infections and vector competence

WNV strain WNV02-1956 was originally isolated from an American crow in New York State in 2005 and passaged once on mammalian cells (Vero; ATCC CC1-81) and once on *Aedes albopictus* mosquito cells (C6/36, ATCC CRL-1660). After an additional passage of 72 hours on C6/36 cells, supernatants and cells from infected cultures were mixed 1:1 with defibrinated bovine blood (HemaResources, Inc, Aurora, OR) plus a 2.5% final sucrose concentration. 7-day old female mosquitoes were deprived of sucrose for 12–16 hours and offered a porcine sausage casing filled with the bloodmeal mixture. Following 1 hour, mosquitoes were sedated with CO_2_ and fully engorged mosquitoes were transferred to 0.6 L cartons and maintained at 27°C for experimental testing. Infection, dissemination, and transmission rates were determined as previously described [14] on days 7 and 13/14 post-feeding. 25–50 mosquitoes/timepoint/group/experiment were sedated and legs were removed and placed in 1 ml mosquito diluent [MD; 20% heat-inactivated fetal bovine serum (FBS) in Dulbecco’s phosphate-buffered saline (PBS) plus 50 μg/ml penicillin/streptomycin, 50 μg/ml gentamicin, and 2.5 μg/ml Fungizone]. Mosquitoes were allowed to expectorate for approximately 30 minutes into capillary tubes filled with FBS plus 50% sucrose (1:1), at which time the mixture was ejected into 0.3 ml MD. Mosquito bodies were then placed in individual tubes with MD. All samples were held at -80°C until tested. Bodies, legs, and salivary secretions were processed and screened by duplicate plaque assay on Vero cells to test for infection, dissemination, and transmission, respectively. Data were analyzed using GraphPad prism 4.0 and rates were compared with Pearson’s chi-squared tests.

### Results and discussion

Vector competence experiments were completed three times and experimental data were combined following confirmation of both equivalent WNV bloodmeal titers and infection rates of genetically similar groups. Variation among dissemination and transmission rates was observed, yet similar trends among individual populations were evident. Mean bloodmeal titers were 9.2 log_10_ WNV/ml. Although this dose mimics peak viremia of some bird species, particularly Passeriformes [15], it is on the high end of what a mosquito would encounter in nature and therefore explains the relatively high infection and dissemination rates measured in all groups (Table 1). Despite this, some differences in infection and dissemination were identified. Among parental populations, *Cx. quinquefasciatus* were the most susceptible, with 100% infection at both timepoints, significantly higher than both *Cx. p.* f. molestus and *Cx. p.* f. pipiens (chi-squared, p < 0.05; Table 1). Although some small differences were identified on day 7, dissemination rates among parental populations were equivalent by day 13/14. As with infection, transmission rates were highest among *Cx. quinquefasciatus*, and this difference was significant relative to both other colonies by day 13/14 (chi-squared, p < 0.05; Table 1). *Cx. quinquefasciatus* have been shown previously to be highly competent WNV vectors [16,17], yet the increased competence relative to other species demonstrated here is likely to be population-dependent [18].

**Table 1:** **Vector competence of *Culex *** **mosquitoes for WNV02 following feeding on infectious bloodmeals**

**Population (female x male)**	**Day**	**% Infected**^**1**^	**% Infected disseminating**	**% Infected transmitting**
***Cx. p *** **f pipiens (CxP)**	7	89.3 ^**M↑ Q↓**^	89.6 ^**Q↑**^	3.0 ^**M,Q↓**^
	13/14	83.6 ^**Q↓**^	98.4	34.4 ^**Q↓**^
***Cx. quinquefasciatus *** **(CxQ)**	7	100 ^**M,P↑**^	69.3 ^**P↓**^	24.0 ^**P↑**^
	13/14	100 ^**M,P↑**^	95.8	63.4 ^**M,P↑**^
***Cx. p *** **f molestus (CxM)**	7	69.6 ^**P,Q↓**^	77.1	16.7 ^**P↑**^
	13/14	93.1	90.0	37.5 ^**Q↓**^
**CxM x CxP**	7	89.3 ^**M↑**^	71.6 ^**P↓**^	13.4
	13/14	76.0 ^**M,P↓**^	93.0	57.9 ^**M,P↑**^
**CxP x CxM**	7	93.3 ^**M↑**^	70.0 ^**P↓**^	7.1
	13/14	94.6 ^**P↑**^	97.1	61.4 ^**M,P↑**^
**CxM x CxQ**	7	100 ^**M↑**^	64.0	8.0 ^**Q↓**^
	13/14	100	94.7	62.7 ^**M↑**^
**CxQ x CxM**	7	100 ^**M↑**^	58.7	5.3 ^**Q↓**^
	13/14	100	93.9	66.1 ^**M↑**^
**CxP x CxQ**	7	100 ^**P↑**^	69.3 ^**P↓**^	2.7 ^**Q↓**^
	13/14	100 ^**P↑**^	97.3	62.7 ^**P↑**^
**CxQ x CxP**	7	100 ^**P↑**^	86.7 ^**Q↑**^	18.7 ^**P↑**^
	13/14	100 ^**P↑**^	100	80.3 ^**P,Q↑**^

^1^65–75 total mosquitoes tested/population/timepoint.

^**M,Q,P**^denotes significant differences in populations (chi-squared, p < 0.05) relative to *Cx. p* f molestus*, quinquefasciatus,* or *p* f pipiens*,* respectively. Arrows denote direction of change relative to control. Parental populations are compared to one another and hybrid populations are compared to corresponding parentals.

**Figure 1:**
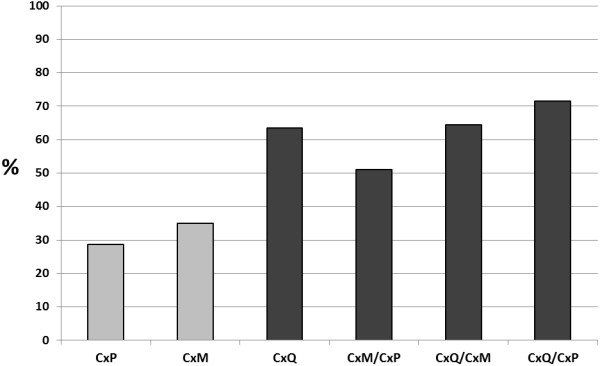
**Percent of WNV-exposed *Culex *mosquitoes transmitting at day 13/14 post-feeding.***Cx.p f pipiens* (CxP), *Cx.p f molestus* (CxM), *Cx. quinquefasciatus* (CxQ), as well as hybrid progeny of these lines were tested. Different shades represent statistically significant differences (chi-squared, p < 0.05).

Assay of hybrid mosquitoes demonstrated that the presence of *Cx. quinquefasciatus* signature increased susceptibility to infection, with 100% infection rates measured in all hybrid groups derived from *Cx. quinquefasciatus* parentals (Table 1). Additional effects of hybridization on infection were measured with crosses of *Cx. p.* f. molestus and *Cx. p.* f. pipiens, with rates generally mimicking the more susceptibility parental strain; yet the most significant effects of hybridization were measured with transmission rates. Specifically, the percent of infected hybrid populations transmitting by day 13/14 was either equivalent to the parental population with the higher measured transmission rate or significantly higher than both parental populations (chi-squared, p < 0.05; Table 1). Overall WNV transmission potential was evaluated by contrasting percent exposed transmitting among populations (% infection *% transmission; Figure 1). These results clearly depict the increased transmissibility of WNV-exposed hybrids, for which *Cx. quinquefasciatus* transmission phenotype dominated in crosses with other species; and a synergistic effect on transmission is observed with crosses between bioformes of *Cx. pipiens* (Figure 1). Anthropogenic and environmental changes are likely to continue to drive alterations to the extent and distribution of hybrids, which could lead to significant shifts in WNV competence among *Culex* populations. Variation in land use is significantly correlated to alterations in WNV, and urbanization not only increases environmental favorability for *Cx. pipiens,* but also is likely to increase the potential for hybridization between bioforms [19,20]. In addition, recent reexamination of hybridization zones demonstates *Cx. quinquefasciatus* genetic signature as far north as New York State [5]. A complete assessment of the vectorial capacity of individual hybrid populations, including evaluation of bloodfeeding behavior and survival, is required to fully characterize the effect on WNV transmission potential, yet these results suggest that the degree of hybridization among *Culex pipiens* complex mosquitoes is likely to be an important factor in WNV epidemiology.

## Competing interest

The authors declare that they have no competing interests.

## Authors’ contributions

ATC directed experiments, analyzed data and wrote the manuscript. PAC carried out experiments and analyzed data. LDK directed and conceived the study. All authors read and approved the final manuscript.
